# The Role of Emoticons in the Comprehension of Emotional and Non-emotional Messages in Dyslexic Youth – A Preliminary Study

**DOI:** 10.3389/fpsyg.2021.693287

**Published:** 2021-08-05

**Authors:** Ewa Leśniak, Szczepan J. Grzybowski

**Affiliations:** Faculty of Management and Social Communication, Institute of Applied Psychology, Jagiellonian University, Kraków, Poland

**Keywords:** reading, verbal communication, nonverbal communication, computer-mediated communication, emojis

## Abstract

The study explored how well-dyslexic youth deals with written messages in an environment simulating popular social network communication system. The messaging systems, present more and more in pandemic and post-pandemic online world, are rich in nonverbal aspects of communicating, namely, the emoticons. The pertinent question was whether the presence of emoticons in written messages of emotional and non-emotional content changes the comprehension of the messages. Thirty-two pupils aged 11–15 took part in the study, 16 had a school-approved diagnosis of dyslexia and were included in the experimental group. Sixteen controls had no diagnosed disabilities. Both groups viewed short messages of four types (each including seven communicates): verbal-informative (without emoticons and emotional verbal content), verbal-emotive (without emoticons, with emotional verbal content), emoticon-informative (including emoticon-like small pictures, but without emotional content either verbal or nonverbal), and emoticon-emotive (with standard emoticons and including verbal-emotional content). The participants had to answer short questions after quick presentation of each message that tested their comprehension of the content. RTs and accuracy of the answers were analyzed. Students without dyslexia had shorter response times to the questions regarding all types of messages than the dyslexic participants. The answers of the experimental group to the questions about the emoticon-informative messages were less correct. The study pointed tentatively to the beneficial role of emoticons (especially the nonstandard, i.e., of non-emotional kind) in reading short messages with understanding.

## Introduction

Of all the learning disabilities developmental dyslexia is the most common, with prevalence rate up to 17% of the population (Shaywitz, 1998) and many of the school children undiagnosed (Barbiero et al., 2012). It is also a source of potentially long-term behavioral, emotional, and psychosocial problems, especially in adolescents (Singer, 2005; Ingesson, 2007; Eissa, 2010). Dyslexia is characterized by poor accuracy and/or fluency in reading, which, alongside poor spelling and decoding abilities, directly impact reading comprehension. According to one of the most influential theories on the causes of dyslexia at its roots is deficits in phonemic access, manipulations, and retrieval (Démonet et al., 2004; Eissa, 2010; Moura et al., 2017; Peters et al., 2020). Effectively, children have difficulties in perception or awareness that words are made up of small, distinctive units that have the potential to differentiate word form and its meaning. These units are long-term representations of higher order than their singular modality-based (auditory, visual, and sensory) counterparts (Démonet et al., 2004). Since phonological (phoneme-based) processes closely relate to the act of hearing and speaking, the deficits can be especially pronounced in reading, which entails awareness that a written word’s units, i.e., letters represent the speech sounds and that they both relate to phonemes. In fact, the phonological awareness is the best single predictor of successful reading (Brady and Shankweiler, 2013). Those phonological working memory deficits have been shown to adversely affect executive functions, such as inhibitory control and selective attention in school children (Barbosa et al., 2019). Indeed, there is some data that developmental dyslexia could be related to more general problems in higher-order cognitive mechanisms like executive attention and multimodal working memory (Varvara et al., 2014).

Dyslexia creates obvious problems in school, but also in personal and social spheres, where adolescents may feel the most vulnerable. Since many social contacts at the current time consist of writing and reading short communications, and this is especially true for the adolescents taking advantage of social media (Valkenburg and Peter, 2011; Oprea and Stan, 2012), it is worthwhile to investigate how young dyslexic people perform while reading short messages of various kinds and how the factors present in online messaging systems affect the performance. The main goal of the present study then is to evaluate reading comprehension of dyslexic youth faced with messages similar to the ones used in popular social networking communication systems in relation to the content of the communicates. It is novel in its approach of exploring reading comprehension in dyslexic youth on the basis of short online messaging. From theoretical standpoint, it could also point out the significant aspects of digital written text perception in general, with a special focus on its nonverbal elements, which are closest and most “natural” counterparts of nonverbal speech units (i.e., facial expressions, emblems, and gestures).

Indeed, one of the most distinctive characteristics of the online messaging systems [or computer-mediated communication (CMC)] is the presence of nonverbal “aids” or cues to the word-based communicates, i.e., the emoticons. Emotional icons (emoticons) are graphic signs that often supplement verbal messages in CMC (Dresner and Herring, 2010) and they perform nonverbal functions in such communication (Lo, 2008). Essentially, they are paralinguistic cues of expressing emotional meaning (Aldunate and González-Ibáñez, 2017), originally developed and used in CMC for the lack of natural means of expressiveness (i.e., face expressions). They are used to express not only emotions and humor, but also to strengthen the verbal contents of the message while impacting its interpretation (Derks et al., 2008a,b). The latter function seems of importance, because it is a less obvious one and could serve to accentuate or better convey strictly informative (non-emotional) contents of the message (e.g., by presenting graphically the most important, content-wise, element of the message). Such emoticons are called “nonstandard” in the present study. Furthermore, emoticons have been described as conveying specific aspects of the speech acts, like user’s intentions (Dresner and Herring, 2010; dos Reis et al., 2018). In broader terms then, emoticons can serve as mediums of illocutionary force. Illocutionary acts are utterances, by which we state, question, command, or promise (Searle, 1969). Because emoticons function in such a wide array of ways, they can have great importance in the proper comprehension of written messages in CMC. In fact, the main rationale behind the present study is their apparent role in enhancing the comprehension of the messages in terms of accuracy of emotions, intentions and attitudes perception (Lo, 2008), clarification of sarcastic or literal meaning (Filik et al., 2016), and user-reported reduction of discourse ambiguity (Kaye et al., 2016). Moreover most young people born after 1980 (from the so-called Millennials generation) are well versed in emoticon use and depend heavily on them in their daily exchanges of written messages (Krohn, 2004).

Fundamentally, emoticons serve as prompts for or reinforcements of both emotional and strictly informative contents of the written communication. Their purpose is to make one’s message as understandable as possible, especially in relation to those elements that are of particular significance to the sender (emotional or non-emotional). Since emoticons are nonverbal in nature, they can be of potentially substantial help in written message comprehension for people with poor reading ability. In the case of dyslexia, these graphic signs could provide non-phonemic strengthening elements enabling readers to achieve better comprehension of the message. There is some data regarding the fact that dyslexic people consciously encode word-like stimuli (pseudowords) differently than controls (attenuated late brain responses), whereas there is no such difference while encoding simple graphic symbols (Schulte-Körne et al., 2004). It is worthwhile then to examine the role and potential benefits of emoticons in reading comprehension in dyslexic youths, who are well acquainted with them and who depend on CMC in their daily lives, especially in the present day’s pandemic and post-pandemic situation, which forces more social isolation and online-only contacts. In the present exploratory and preliminary study, we are interested if and how standard (emotional) and nonstandard types of emoticons help young students with dyslexia in the understanding of both emotional and non-emotional verbal online messages. We expect overall worse performance (reading times and accuracy) in dyslexic participants as compared with the controls, with some beneficial effects of emoticons on the messages comprehension observed especially in the experimental group.

## Materials and Methods

### Advanced Preparation

In order to gather more information on the students of the age group and their communication preferences, we used two complementary data sources. One was special national research on young people (National Research Institute of Poland NASK report; Bochenek and Lange, 2019) and the other were the current study’s conversations and interviews with school children. The short interviews preceded the day of the experiment and they were conducted at the same school as the experimental sessions. This was facilitated by school’s counselors, who had access to the dyslexia diagnosis of the pupils. The researchers recruited 34 students who participated in the interview stage in preparation for the study (dyslexic *N* = 17). Of these, 32 took part in the subsequent study. We also contacted 15 age-matched students attending many different schools, who did not take part in the study, *via* e-mails and smart phone messages prior to the interviews. During the conversations, questions regarding preferred social media platforms as well as the main purpose and characteristics of their usage (see below for details) were asked. This was done in order to confirm the more robust data from the NASK report.

The interviews conducted in preparation for the study supported the data from the NASK report in terms of the importance of communication *via* social media for adolescents. According to the report, only 0.2% of primary school respondents declared having no profile on social media sites and 77.8% of respondents stated that they use Facebook as their favorite social media platform. Based on the data, it was decided that an adaptation of Facebook Messenger would be used as a basis for materials to be presented in the study (ecological validity purposes). The conversations with the age group confirmed that adolescents are very familiar with the Messenger. The respondents highlighted the fact that they used the application primarily for social purposes. Regardless of the frequency of active usage of the Messenger to send messages, most of the young people were subjected to its passive influence, i.e., getting messages from other people. The pupils were asked specific questions regarding topics of conversations and also emoticons most commonly used in the Messenger. It was concluded that adolescents communicate by the means of the Messenger application to talk about daily life, school, current events, nearby future, and to arrange meetings and dates. Although the use of emoticons was dependent on personal preferences, the most often used ones were those related to emotional states, enrichment of expression, or replacement of words.

### Participants

Thirty-two Polish primary school pupils aged 11 to 15 (mean = 13.28, *SD* = 1.05) took part in the study (females *N* = 15). Sixteen participants (mean age = 12.81, *SD* = 1.22, females *n* = 8) were qualified to the experimental group on the basis of dyslexia diagnosis, and 16 participants (mean age = 13.75, *SD* = 0.57, females *n* = 9) made part of the control group. Dyslexia diagnosis was based on the headmaster’s and school counselors’ declaration stating that particular children had certificates from a psychological and pedagogical counseling center. Children diagnosed with other specific developmental disorders of scholastic skills were not included in the experimental group. Students in the control group had no diagnosed impairments.

The research was conducted in accordance with the ethical demands, provided and approved by the Commission on the Ethics of Scientific Research at the Jagiellonian University Institute of Applied Psychology. Before the experiment, signed consent forms were also obtained from parents or legal guardians of the participants.

### Materials

We devised the messages on the basis of the data sources (NASK report and the preceding interviews) and implemented them in a picture frame that simulated the Messenger’s graphical user interface.

**Figure 1:**
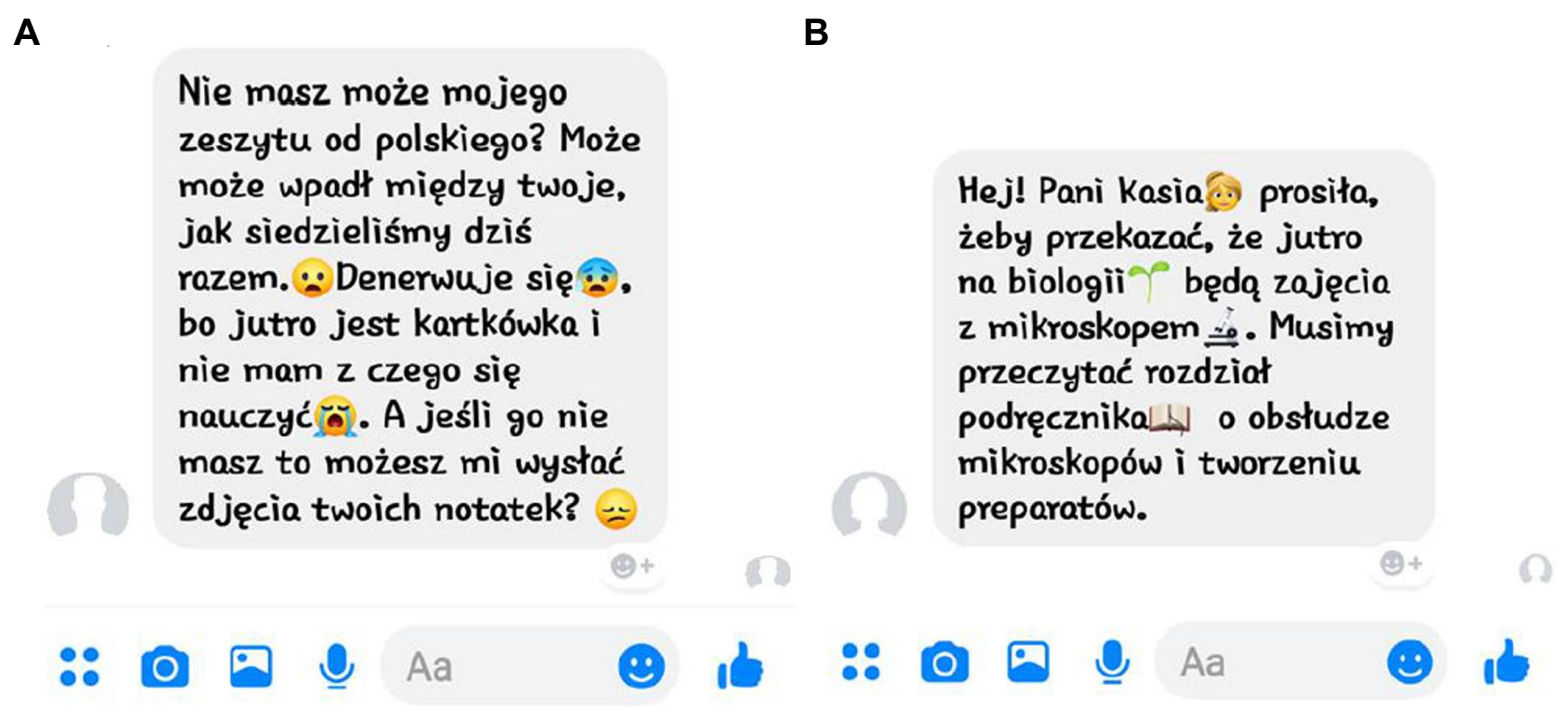
Two sample messages with emoticons used in the experiment. **(A)** Emoticon-emotive (E-E) message, translated from Polish: Do you have my Polish class notebook? Maybe it ended up in your things, while we were sitting together. (emoticon) I am nervous (emoticon), because we have a test tomorrow and I cannot prepare without it (emoticon). If you do not have it, would you send me pictures of your notes? (emoticon). **(B)** Emoticon-informative (E-I) message, translated from Polish: Hey! Mrs. Jones (emoticon) asked to tell others that tomorrow in biology class (emoticon) we will work with microscopes (emoticon). We need to read a chapter from the manual (emoticon) on the use of the microscopes and the preparation of the samples. Message icons and layout © Facebook.

The messages were divided into four groups. Every group consisted of seven separate messages (each containing on average four sentences, minimum three, maximum six, made up of content words resembling the youth lexicon as closely as possible, with no difficult or infrequent words present). The first group was called verbal-informative (V-I) and its messages lacked emotional content and any emoticons (sentences and questions on neutral topics regarding school, house chores, and extra-curricular activities); the second group, verbal-emotive (V-E), lacked emoticons but possessed emotional verbal content (sentences and questions on significant, stressful, or exciting topics); the third was called emoticon-informative (E-I) and it included nonstandard emoticons (signs illustrating objects, events, and persons) and the verbal content of its messages was non-emotional; and lastly, the fourth group, emoticon-emotive (E-E), included standard emoticons (expressing various emotional states) and emotional verbal content. Each message within the two emoticon groups contained four emoticons. Figure 1 illustrates samples of two messages used in the experiment.

In order to test the comprehension of the written messages from each group, 28 sets of questions were prepared. It was decided to posit two types of test questions, both strictly relating to the content of each message: two single-choice questions (with four possible answers) and two yes or no questions (four questions for every message in total). The two types of questions were presented for every message shown. Such design let us examines the understanding of the conveyed content, and not the short-term memorization of a specific word used in a message. The questions referred to crucial content that could be important to the recipient in the case of receiving similar types of messages in real life. Here, we present two examples of the questions:

*Were there any beverages on the shopping list? Yes/no*.

*The sender of the message needs the guitar because: (1) he will play a gig*, *(2) he has a guitar class scheduled*, *(3) he will participate in a family get-together*, and *(4) he just wants to play it*.

Additionally, Edinburgh Handedness Inventory (Oldfield, 1971) was administered.

### Procedure

The procedure was programmed in the PsychoPy2 software (Peirce et al., 2019). The experiment was conducted in classrooms which were made available by headmasters and school counselors. Firstly, the participants were asked to fill out the consent form and then Edinburgh Handedness Inventory. Next, the procedure was run on a notebook, starting with instructions that detailed the task ahead and introduced the training session, in which two sample messages (not present in the actual experimental run) with the standardized sets of questions were presented. After the training session, the participants had the opportunity to ask questions if anything was unclear to them. Then, the main experimental session began. Each participant was faced with all the messages in random order from four groups described in the materials section. Each message was presented for 20 s on the computer screen and immediately after each presentation a set of four questions (two multiple-choice questions and two yes or no questions) was presented randomly one at a time. There was no time limit for giving answers. Reaction time and correctness of the answers were registered. The whole procedure took approximately 30 min. At the end, the participants received words of appreciation and were free to leave.

## Results

The statistical analyses were conducted in the Statistica 13 and SPSS 26 software packages. Edinburgh Handedness Inventory revealed that most of the participants were right handed (*n* = 30) and a few left handed (*n* = 2). The main experimental session analyses were based on one independent variable with four levels (type of message: verbal-informative V-I, verbal-emotive V-E, emoticon-informative E-I, and emoticon-emotive E-E) and two dependent variables (RTs – mean from the single choice and yes/no questions to each type of message and the correctness of answers – mean sum of the points of the four questions).

In the first part of the analysis, distributions of the RT and accuracy of the answers were tested. The *W* Shapiro-Wilk test was conducted for this purpose. A normal distribution was revealed for variables: RTs of the answers to the questions on V-E and V-I messages as well as E-I messages. Other variables turned out not to have normal distribution. Further inspection of the accuracy scores distributions revealed that the data were negatively skewed. Log10 transformation attempt at normalization did not change the skewness of the distribution. Therefore, the differences in the correctness variable were analyzed with the nonparametric test.

### Reaction Times of the Answers

**Figure 2:**
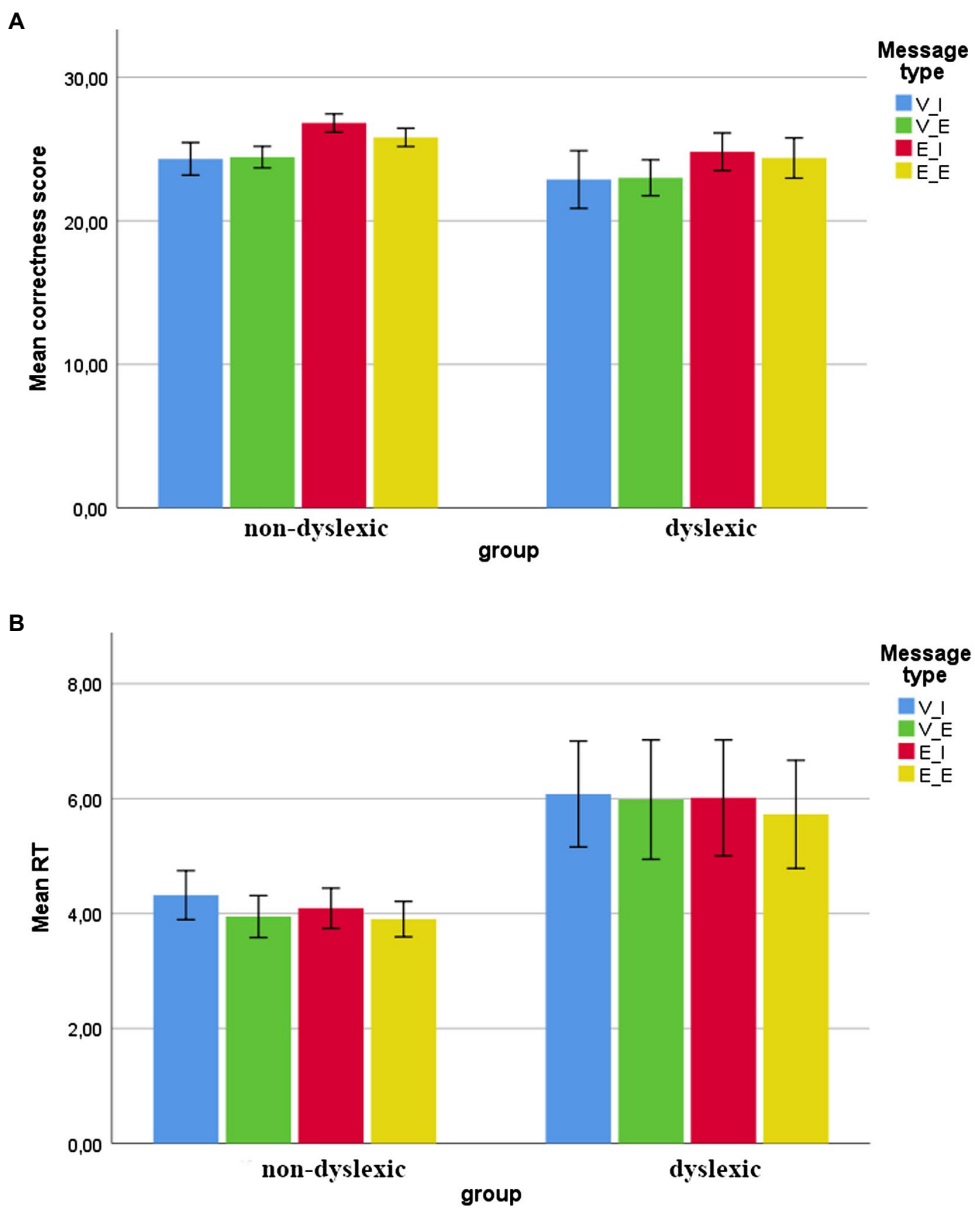
Mean scores for all the message types in two groups with standard error bars. **(A)** Mean correctness score **(B)** mean RT (in seconds). V-I, verbal-informative message; V-E, verbal-emotive; E-I, emoticon-informative; and E-E, emoticon-emotive.

Since the present study was exploratory and preliminary in nature, it is worthwhile to emphasize the descriptive statistics first in order to indicate general trends. In the case of the RTs, the quickest answers were given to emoticon-emotive (E-E) messages in both groups (dyslexic group mean 5.72 s, *SD* = 1.88, min 3.43, max 10.85; non-dyslexic group mean 3.9, *SD* = 0.61, min 2.94, max 4.84) and the longest to verbal-informative (V-I) messages, again in both groups (dyslexic mean 6.07, *SD* = 1.84, min 3.9, max 11.16; non-dyslexic mean 4.31, *SD* = 0.85, min 3.05, max 5.92). Figure 2 illustrates the descriptives (see Figure 2B for the RTs).

In order to compare the groups in terms of the RTs of answers having similar normal distribution, analysis of variance was conducted. The results showed statistically significant differences between the control and experimental groups, *F*(3.28) = 4.36, *p* < 0.05. In order to evaluate the differences of particular variables between the groups, Bonferroni’s *post-hoc* test was carried out. All the variables turned out to be significant (*p* < 0.05). The results indicate getting significantly shorter reaction times of answers to all the types of messages examined in the group of participants without dyslexia. Table 1 presents the differences in RTs between the groups.

**Table 1:** Mean RT of answers to three types of messages in two groups with significance levels of Bonferroni’s tests.

Message type	Dyslexia group	No dyslexia group
V-I	6.08^*^	4.32^*^
V-E	5.98^*^	3.95^*^
E-I	6.01^*^	4.09^*^

*V-I, verbal-informative message; V-E, verbal-emotive; and E-I, emoticon-informative*.

* *p* < 0.01.

Also, the RTs of answers to questions regarding the verbal-emotive (V-E) messages were overall faster than to verbal-informative (V-I) ones (mean RTs 4.96 < 5.19, *p* = 0.02) for all the participants (irrespective of group). In other words, the answers to verbal-only messages with emotional content were given faster than to those without emotional content.

### Correctness of the Answers

As in the case of the RTs, we emphasize the descriptives as indicators of general trends. The most accurate answers were given to emoticon-informative (E-I) messages in both groups (dyslexic group mean 24.81, *SD* = 2.61, min 20, max 28; non-dyslexic group mean 26.81, *SD* = 1.27, min 24, max 28) and the least accurate to the verbal-informative (V-I) ones, again in both groups (dyslexic mean 22.88, *SD* = 4.01, min 14, max 27; non-dyslexic mean 24.31, *SD* = 2.27, min 20, max 28). Figure 2 illustrates the descriptives (see Figure 2A for accuracy scores).

The lack of normal distribution of the correctness variable resulted in conducting nonparametric Mann-Whitney *U* test. The results indicate the significance (*U* = 64.5, *p* < 0.05) of the correctness related to one type of message, i.e., having informative content with (nonstandard) emoticons (E-I). Answers of the control group turned out to be more accurate comparing to the ones given by the participants from the experimental group. Table 2 shows the differences.

**Table 2:** Mann-Whitney U test statistics (with the continuity correction) of the correctness of answers between groups for the four types of messages used in the study.

Message type	*U*	*Z*	*p*	*Z* (correct)	*P*	*N* important (Dyslexia group)	*N* important (No dyslexia group)
V-I	112.0000	−0.58	0.559	−0.59	0.554	16	16
V-E	64.0000	−1.63	0.101	−1.68	0.092	16	16
E-I	64.5000	−2.37	0.017	−2.42	0.015	16	16
E-E	90.0000	−1.41	0.157	−1.44	0.148	16	16

*V-I, verbal-informative message; V-E, verbal-emotive; E-I, emoticon-informative; and E-E, emoticon-emotive*.

To look for differences in the accuracy of the answers to different messages within the groups, Wilcoxon rank sum test was conducted. In dyslexic group, the most accurate answers were given to informative messages with nonstandard emoticons (E-I) and those differed significantly with purely verbal-informative messages (V-I), *W* = 96.00, *p* = 0.005, and verbal-emotional messages (V-E), *W* = 89.00, *p* = 0.002. More accurate answers were also given to emotional messages with emoticons (E-E) as compared to emotional ones without any emoticons (V-E), *W* = 23.00, *p* = 0.03. In non-dyslexic group, the higher accuracy of the answers to E-I messages was even more pronounced, with significant differences as compared to V-I messages, *W* = 6.00, *p* = 0.006, V-E messages, *W* = 12.00, *p* = 0.003, and even E-E ones, *W* = 19.50, *p* = 0.03. There were also differences observed between (more accurate) answers to E-E messages and V-I ones, *W* = 56.00, *p* = 0.03, as well as between E-E messages and V-E ones, *W* = 9.00, *p* = 0.009.

## Discussion

The aim of the preliminary study was to explore whether the presence of two types of emoticons (traditional and nonstandard) within emotional and non-emotional verbal messages helps with the comprehension of their contents in dyslexic youth. The reading disability is proven to influence adversely young people’s growth and wellbeing, both in social and personal domains (Terras et al., 2009; Eissa, 2010; Glazzard, 2010; Dahle et al., 2011) with children’s behavior and personality being negatively affected as well, impacting their quality of life (Gagliano et al., 2014; Huang et al., 2020), which in turn may lead to such severe problems as depression and suicidality. This necessitates coping programs based on whole-school support systems (Firth et al., 2013) or special compensation tools, e.g., software with user-driven functionalities aiding reading comprehension and fluency (Rodriguez-Goncalves et al., 2021), especially taking into account the fact that many teachers lack the strategies to evaluate and intervene in dyslexic students (Leite, 2012; Ryder and Norwich, 2019). In an ever isolated pandemic and post-pandemic world that depends more and more heavily on CMC, whether for social, educational, or personal purposes, it is especially important to study what are the possible beneficial factors in reading comprehension for dyslexic young people. That is why we decided to look into the most characteristic, yet constantly expanding and developing aspect of the CMC, i.e., emoticons. Emoticons have evolved from simple graphic signs relating to smiles, frowning, or expressions of sadness (imitations of facial expressions) to illustrations of complex concepts of significance for the sender (objects, persons, events, and situations; Dresner and Herring, 2010). They also became less typographic and more human or reality-based in nature and as such are sometimes called *emojis* (Aldunate and González-Ibáñez, 2017). In the present study, we decided to employ emoticons (emojis) that belonged to two main types: traditional, emotion-based and nonstandard, information (object or person)-based ones.

We compared the comprehension of the written messages with or without emoticons on the basis of reaction times and accuracy (correctness) of the answers given in experimental (dyslexic) and control groups of age and sex matched participants. We expected poorer overall performance of the experimental group, which was confirmed as far as the RTs were concerned. Longer RTs in dyslexic youth could point to the problems in reaching the proper information through the working memory (first questions on the contents of the messages were asked immediately after 20 s of message presentation), which could be due to the impaired comprehension of time-restricted text presentation. Alternatively, the effect could be seen as the result of trouble in the encoding of the (written) questions themselves, either on the basis of their only verbal (phonological) elements or processing of fast and rapidly changing stimuli. The latter aspect could relate to the speed processing hypothesis of deficits in dyslexia (Tallal, 1980). However, the alternative explanation seems a less probable one, since the questions were explicitly designed to be as simple and straightforward as possible tests of content comprehension (single choice and yes/no types of questions, no time limit for an answer). The overall high performance in the accuracy of the answers (see below) would attest to that. The RT effect can be seen as a point in favor for providing more time during educational process for dyslexic youth, including written state examination.

The general trends as indicted by descriptive statistics point to the fastest answers being given in response to emoticon-emotive messages in both groups. This possibly relates to the main effect of quickest RTs to the verbal messages rich in emotional content (see below), but also could be seen as a tentative point in favor of traditional (face-expression based) emoticons as the most common and natural paralinguistic cues in CMC that could help in written message encoding (Aldunate and González-Ibáñez, 2017). Conversely, messages lacking any nonverbal cues and without emotional content (verbal-informative only) seem to be the hardest to process in CMC (having the lowest accuracy scores in both groups as well, see Figure 2A).

We also noted a general main effect of emotional content of strictly verbal messages on the RTs, with overall (irrespective of group) shorter RTs to the emotive messages as compared to the informative ones. As such, it is marginally interesting in the context of the present study, but it possibly showcases a well-researched aspect of preferential processing of emotional stimuli, pictorial or verbal alike, evidenced strongly even on brain activity measures (for a review on image-based studies, see Olofsson et al., 2008 and on word-based studies, see Citron, 2012). It could also relate to the specific aspect of better memorization of autobiographical content rich in emotional elements (Christianson and Safer, 1995), since the messages used in the study had social and personal overtones and they related to the episodes of everyday life commonly experienced by young people.

We have observed differences in relation to accuracy of the answers between the experimental and control groups. Significantly more accurate answers were given by non-dyslexic participants to messages with emoticons and of informative content only. The graphic signs in those messages were of nonstandard type, i.e., small pictures of objects or persons that were also expressed verbally. These newer kinds of emoticons (which might as well be called “infoicons”) repeat or reinforce content already conveyed. They seem of particular use for quickly grasping the meaning or better encoding of the content to be recognized within the next minute. Non-dyslexic people seem to make the best use of such graphic reinforcement of the verbal message content (also as compared to all the other types of messages within that group). However, on the basis of within-subject analysis, we tentatively observed the benefits of exactly that kind of emoticons for dyslexic people as well. Trends indicated by descriptives (see Figure 2A) attest to that as well. The analyses and general trend observations showed that the answers to the informative messages with nonstandard emoticons (E-I ones) were the most accurate ones and they differed significantly with both types of messages (informative and emotional in content) that lacked emoticons. Possibly then, it is the nonstandard emoticons that are of most benefit to the individuals with reading impairment, since they are purely nonverbal (non-phonemic) signs that serve as graphic transcripts of verbal content, and thus help in the message comprehension. As such, they could be implemented into educational programs and online studies as aids in reading comprehension tasks.

Traditional emoticons (conveying basic emotional states, such as happiness, sadness, and surprise) on the other hand could be seen as more complex in nature since they do not duplicate the content, but rather add nuanced interpretation (or intentions) of the sender to it. What is more, although they too are nonverbal functionally, they could be seen as quasi-nonverbal elements (Lo, 2008), as the additional content to the verbal series (verbal cues). Such an interpretation should be approached cautiously, because there was no significant difference in the accuracy of answers between messages with traditional and nonstandard emoticons in the experimental group (it was present however in the controls). Interestingly, there is some evidence that people with dyslexia have visual attention deficits that relate to general visual domain, rather than to strictly verbal one (Lobier et al., 2012).

It is also worth noting that the overall accuracy-based performance of the dyslexic group was high (only one significant difference between the groups). This can point to the fact that the task was very simple indeed, or alternatively that the CMC which was simulated by design in the study’s procedure, is a very natural and enabling environment, especially for young people, including those with reading disabilities, even though they require more time to react to the written messages (see RT effects described above).

The overall trend of higher accuracy of the answers to messages rich in both kinds of emoticons as compared to the strictly verbal messages in both groups is also worth mentioning. This general tentative effect seems to confirm special role of emoticons in CMC in enhancing the comprehension of the written text, possibly by strengthening the verbal content of the message (Derks et al., 2008a) or clarifying its ambiguity (Kaye et al., 2016). The most important aspect that the study points to is their potential benefit for dyslexic students as aids in educational process and social interactions alike (obviously, since the claim is based on the results of a simple preliminary study it should be treated very cautiously). In a world that depends on CMC more and more in educational, professional, and personal spheres, the need to understand and pinpoint crucial aspects of written content comprehension for people with reading impairments is a pressing matter. Future research on larger samples should focus on short yet condensed (content-wise) messages and text excerpts in detailing the role of various kinds of emoticons (standard vs. nonstandard) with different degrees of complexity (colors, shapes, and animations) and determining the most beneficial type of paralinguistic cue for written content comprehension (with strict control for communication patterns of young people). The new, nonstandard object-based emoticons reinforcing the verbal content by essentially doubling it, bearing close resemblance to reality (*emoji* class) as present in the most popular social network messaging system (duplicated in the present study) seem the most promising or interesting of the aids.

Lastly, limitations of the study ought to be mentioned. Since the study was explorative and preliminary in nature, the sample size of the participants was small, and no prospective power analysis was done to determine the adequate sample. This obviously limits the interpretation of the data and results obtained. Retrospective power analysis was not implemented, since it adds no new information on the statistical tests outside the value of *p* and should be avoided (Lenth, 2001). Furthermore, the score distribution of the correctness variable was not normal and this resulted in conducting nonparametric tests. Both of these factors (the non-normal distribution probably stemming in part from the small sample size) renders the analysis problematic and the interpretations of the results and conclusions based on them should be treated cautiously and only as tentative indicators of the possible effects in the population. The other cause of the non-normal distributions of the correctness results worth mentioning is very low difficulty of the task employed and this in turn resulted in a positive bias (toward the high end of scales) of the scores (negatively skewed distribution). The study design itself could be seen as not optimal for full investigation into emoticons and its impact on the comprehension of various kinds of online messages, since for simplicity and ecological validity purposes it lacked, e.g., emotional text message condition with nonstandard (non-emotional) emoticons (such messages seem rare in real CMC). Furthermore, there are some potential confounding factors that could have had an impact on the results, like initial level of text comprehension, long-term experience with CMC, which should be addressed and controlled in future full-scale research.

Overall, the study obtained some tentative and promising results, which pointed to specific factors of importance in reading comprehension of the students with dyslexia (the nonstandard emoticons, longer times of written message processing). As such merits replication on larger samples and further exploration of the abovementioned aspects related to online written content so commonly accessed by young people nowadays.

## Data Availability Statement

The raw data supporting the conclusions of this article will be made available by the authors, without undue reservation.

## Ethics Statement

The studies involving human participants were reviewed and approved by the Commission on the Ethics of Scientific Research of the Institute of Applied Psychology, Jagiellonian University. Written informed consent to participate in this study was provided by the participants’ legal guardian/next of kin.

## Author Contributions

EL contributed to the study design, the gathering of the data, the analysis and interpretation of the data, and the drafting of the manuscript. SG contributed to the study design, the analysis and interpretation of the data, and the drafting and critical revision of the manuscript. All authors contributed to the article and approved the submitted version.

## Conflict of Interest

The authors declare that the research was conducted in the absence of any commercial or financial relationships that could be construed as a potential conflict of interest.

## Publisher’s Note

All claims expressed in this article are solely those of the authors and do not necessarily represent those of their affiliated organizations, or those of the publisher, the editors and the reviewers. Any product that may be evaluated in this article, or claim that may be made by its manufacturer, is not guaranteed or endorsed by the publisher.
